# Sub‐Nanometer Thick Gold Nanosheets as Highly Efficient Catalysts

**DOI:** 10.1002/advs.201900911

**Published:** 2019-08-06

**Authors:** Sunjie Ye, Andy P. Brown, Ashley C. Stammers, Neil H. Thomson, Jin Wen, Lucien Roach, Richard J. Bushby, Patricia Louise Coletta, Kevin Critchley, Simon D. Connell, Alexander F. Markham, Rik Brydson, Stephen D. Evans

**Affiliations:** ^1^ School of Physics and Astronomy University of Leeds Leeds LS2 9JT UK; ^2^ Leeds Institute of Medical Research St James's University Hospital University of Leeds Leeds LS9 7TF UK; ^3^ School of Chemical and Process Engineering University of Leeds Leeds LS2 9JT UK; ^4^ Division of Oral Biology School of Dentistry University of Leeds Leeds LS9 7TF UK; ^5^ Institute of Organic Chemistry and Biochemistry AS CR 166 10 Praha 6 Czech Republic

**Keywords:** 2D, catalysis, gold nanomaterials, nanoenzymes, sub‐nanometer

## Abstract

2D metal nanomaterials offer exciting prospects in terms of their properties and functions. However, the ambient aqueous synthesis of atomically‐thin, 2D metallic nanomaterials represents a significant challenge. Herein, freestanding and atomically‐thin gold nanosheets with a thickness of only 0.47 nm (two atomic layers thick) are synthesized via a one‐step aqueous approach at 20 °C, using methyl orange as a confining agent. Owing to the high surface‐area‐to‐volume ratio, abundance of unsaturated atoms exposed on the surface and large interfacial areas arising from their ultrathin 2D nature, the as‐prepared Au nanosheets demonstrate excellent catalysis performance in the model reaction of 4‐nitrophenol reduction, and remarkable peroxidase‐mimicking activity, which enables a highly sensitive colorimetric sensing of H_2_O_2_ with a detection limit of 0.11 × 10^−6^ m. This work represents the first fabrication of freestanding 2D gold with a sub‐nanometer thickness, opens up an innovative pathway toward atomically‐thin metal nanomaterials that can serve as model systems for inspiring fundamental advances in materials science, and holds potential across a wide region of applications.

## Graphical abstract

**Sub‐nanometer gold nanosheets (thickness: 0.47 ± 0.01 nm)** are successfully prepared via a facile aqueous synthesis at an ambient temperature. These nanosheets represent the first free‐standing 2D gold nanostructure with a sub‐nanometer thickness, and demonstrate excellent catalysis performance in the model reaction of 4‐nitrophenol reduction, coupled with remarkable peroxidase‐mimicking activity, which enables a highly sensitive colorimetric detection of H_2_O_2_.

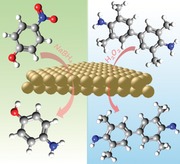

2D nanomaterials with thicknesses of single to few atomic layers, as exemplified by graphene, have stimulated enormous interest, owing to their unique electronic, mechanical, and surface‐related properties that arise from their reduced dimensionality.^1^, ^2^, ^3^ Freestanding ultrathin metal nanostructures have recently become the rising stars of 2D materials, because of their potential for applications in bioimaging, therapy, sensing, and catalysis.^4^, ^5^ For instance, ultrathin 2D noble metal nanomaterials have attracted increasing attention due to their ultrathin nature and 2D morphology. The ultrathin nature leads to high surface area‐to‐volume ratio and abundant exposed catalytically‐active sites.^6^, ^7^, ^8^ The 2D morphology confers a large interfacial area in contact with the substrate compared with either 1D or 3D nanostructures (e.g., nanowire or nanoparticles), which can enhance the interactions between reactants and the surface of catalysts, contributing to high activity.^8^

In view of the fascinating attributes and numerous potential applications of ultrathin 2D metal nanomaterials associated with their unique structural features, it is essential to develop feasible facile and reliable synthesis routes.^2^ However, the production of ultrathin 2D metal nanomaterials, free of a solid substrate, represents a significant challenge, due to the tendency of metal atoms to form a highly isotropic 3D close‐packed crystal lattice.^9^ This natural tendency toward 3D growth can be suppressed by the introduction of confinement to induce anisotropic growth.^4^ To date, a range of synthesis strategies have been utilized to prohibit the free growth of primary metal nuclei and promote 2D anisotropic growth, using a variety of confinement substances, including: 1) surface‐active agents, such as polymers^10^ and gases^9^, ^11^ that selectively bind onto low‐index metal surfaces; 2) templates, e.g., lamellar hydrogels,^12^ graphene and its derivatives.^13^ Currently, however, there is no synthetic strategy that allows ambient wet‐chemical synthesis of free‐standing atomically‐thin 2D metal nanostructures.

In this communication, we report a facile synthetic strategy based on the use of methyl orange (MO) to prepare atomically‐thin gold nanosheets (termed gold nanoseaweed, because of its morphology, color, and aqueous growth). The obtained gold nanoseaweeds (AuNSWs) exhibit high efficiency as heterogeneous nanocatalysts and peroxidase‐mimicking nanoenzymes.

For the synthesis of Au nanoseaweed, aqueous solutions of HAuCl_4_ and Na_3_C_6_H_5_O_7_ (sodium citrate, SC) were sequentially added into an aqueous solution of MO (0.21 × 10^−3^ m) at 20 °C. The resultant solution was kept undisturbed at this temperature for 12 h (see **Figure** **1**a and the Supporting Information). The reaction products were collected by centrifugation, and then washed several times with Milli‐Q water until the supernatant was colorless. The centrifuged pellet was readily re‐dispersed in Milli‐Q water for further characterization.

**Figure 1:**
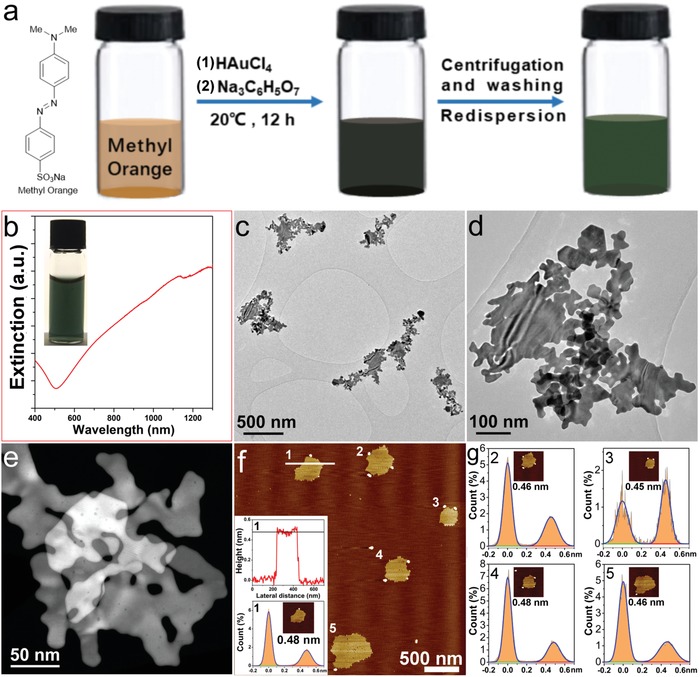
Synthesis and characterization of Au nanoseaweed: a) schematic representation of the synthesis route; b) UV–vis spectrum, with inset showing a digital image of the dispersion; c,d) bright field TEM images at different magnifications; e) dark field STEM image; f) AFM image with inset showing the thickness profile measured at the white line as indicated, and depth histogram of Sample 1; g) depth histograms of Samples 2–5. Heights were obtained from Gaussian fits of histograms extracted from each AuNSW (inset), cropped from pre‐flattened images and further leveled using high‐order plane fits to remove ringing oscillation, together with median filtering to reduce random noise.

The as‐obtained dispersion displays a green–blue color (Inset in Figure 1b) and remains stable under ambient conditions for >15 months. The UV–vis spectrum of the dispersion exhibits a broad extinction band in the region of 500–1300 nm. The lack of a peak around 520 nm (Figure 1b) indicates the absence of isotropic gold nanoparticles.^14^ A representative transmission electron microscopy (TEM) image of the product (Figure 1c) demonstrates the high‐yield formation of 2D nanostructures with each exhibiting a “seaweed‐like shape.” Detailed analysis of TEM images of 20 individual AuNSWs (Figure S3, Supporting Information) reveals that they have similar fractal dimensions, with values within the range 1.69–1.78 (vide supra). Higher‐magnification bright field (BF) TEM images show that the AuNSW exhibits bend contours suggesting flexibility (Figure 1d). The translucent appearance, folded edges, and wrinkles (shown in Figure 1e and Figure S4, Supporting Information) indicate the ultrathin nature of the AuNSW,^15^ which is verified by the thickness measurement using atomic force microscope (AFM). The height profile measured by AFM at the white line in Figure 1f demonstrates the AuNSW is atomically flat, with a thickness of ≈0.48 nm. To minimize the random noise and systematic noise associated with scan lines in AFM measurements (see the Supporting Information), the thicknesses of AuNSWs were carefully determined by fitting Gaussian peaks to depth histograms (inset of Figure 1f,g). The height histogram peaks can be clearly discriminated for each sample, and the analysis demonstrates that the thickness of AuNSW is (0.47 ± 0.01) nm.

The crystal structure of AuNSWs was investigated using HRTEM, selected‐area electron diffraction (SAED) and X‐ray diffraction (XRD). The HRTEM image of AuNSW (**Figure** **2**a) exhibits a sixfold symmetric structure with a lattice spacing of 0.25 nm, consistent with the 1/3 {422} lattice spacing of fcc‐gold.^16^ The SAED pattern in the [111] zone axis (Figure 2b) displays two sets of sixfold symmetric spots, including strong spots (boxed) identified as the allowed {220} Bragg reflection (corresponding to the lattice spacing of 0.144 nm), and weak spots (circled) identified as the forbidden 1/3{422} (corresponding to the lattice spacing of 0.250 nm).^16^, ^17^ Both HRTEM and SAED results suggest the single‐crystalline nature of the AuNSW with a <111> orientation. Therefore, according to the thickness measured by AFM, the AuNSW contains two layers of Au atoms. It is noteworthy that {422} reflections are forbidden in the fcc structure, and only observable for an ultrathin gold (or silver) sample that is atomically flat.^16^, ^18^, ^19^, ^20^ The presence of the forbidden reflection in the AuNSW is ascribed to local regions of incomplete cubic (ABC) packing deriving from the ultrathin nature, and local hexagonal close packing (hcp) in low‐dimensional gold (or silver) nanostructures.^18^, ^20^ The values of fractal dimension of our AuNSWs are close to 1.71 (Figure S3, Supporting Information), suggesting the formation via a diffusion‐limited aggregation pathway.^21^ According to the Gibbs–Thomson theory, dull corners have lower‐energy compared with sharp ones. The corner atoms are inclined to migrate to lower energy states, generating more stable nanocrystals.^22^, ^23^ As a result, the edge and corner structures of AuNSW (Figure 2d–f) display relatively dull and smooth features, which represent the lower‐energy state via the diffusion‐limited growth and contribute to the stability of AuNSWs. Careful observation also reveals the presence of hcp phase (ABAB) at the edges of the AuNSW (indicated by red arrows in Figure 2e,f). This non‐fcc crystalline feature has been acknowledged to be able to stabilize ultrathin gold nanostructures.^24^, ^25^, ^26^ Correspondingly, the XRD pattern of AuNSW (Figure 2c) shows a dominant (111) peak at 38.2°, revealing that <111>‐oriented fcc Au crystals are predominant in the AuNSW sample. In addition to the main Bragg reflections of fcc Au, shoulders at ≈37° and ≈40° respectively can be assigned to the (002) and (101) lattice spacing of an Au hcp phase,^13^ which has been previously reported to occur in ultrathin or small gold nanostructures.^24^, ^25^

**Figure 2:**
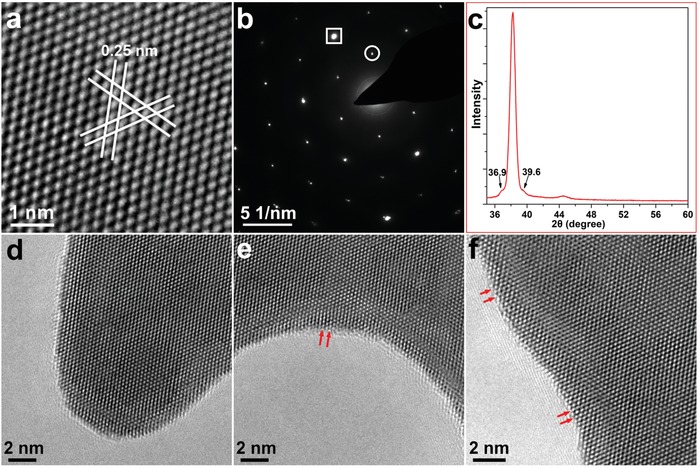
Crystal structures of AuNSWs: a) HRTEM image with spacings between each set of white parallel lines measured to be ≈0.25 nm, corresponding to the 1/3 {422} lattice spacing of fcc‐gold; b) Selected area electron diffraction pattern (SAED) down the [111] zone axis: strong spots (boxed) indexed as the allowed {220} Bragg reflections (corresponding to a lattice spacing of 0.144 nm); weak spots (circled) indexed as the forbidden 1/3{422} reflections (corresponding to a lattice spacing of 0.250 nm); Figure S5 (Supporting Information) shows the TEM image of the region corresponding to SAED in b,c) XRD pattern over a 2θ range from 30° to 60°. Figure S6 (Supporting Information) shows the XRD pattern of AuNSWs over a 2θ range from 30° to 90°; d–f) HRTEM images of the corner and edges. Red arrows indicate some of the HCP structures at the edges.

In order to explore the formation mechanism of the AuNSWs, we carried out simulation and a series of control experiments. First, we performed a control experiment of synthesizing gold nanostructures in the absence of the confining agent MO whilst keeping all other reaction parameters unaltered. The products showed a quasi‐spherical shape (**Figure** **3**a) with a size of (50.8 ± 0.2) nm (inset in Figure S7, Supporting Information), and an absorption peak around 530 nm (Figure S7, Supporting Information), substantiating that the use of MO is critical for yielding 2D nanostructure. We selected MO as a potential confining agent, because MO molecules consist of a rigid aromatic moiety, a hydrophobic section and hydrophilic side groups, and were reported to self‐associate for forming planar stacks in aqueous solution.^27^, ^28^, ^29^ We consider that the planar stacking of MO may play a role in dictating the growth of 2D gold nanostructures. To investigate whether the rigid aromatic moiety or the side groups of MO interact with Au in our system, we performed a control experiment by replacing MO with 2,2′‐Bipyridine, which only possesses a rigid moiety, and has been documented to form 2D self‐assembly in aqueous solution.^30^ Synthesis using 2,2′‐Bipyridine, while keeping other reaction conditions unchanged, yields gold nanosheets (Figure S8, Supporting Information) with SAED showing <111>‐orientation with atomically flat surfaces. Therefore, we infer that MO molecules interact with gold by the rigid moiety (azo‐benzene component) in our reaction system.

**Figure 3:**
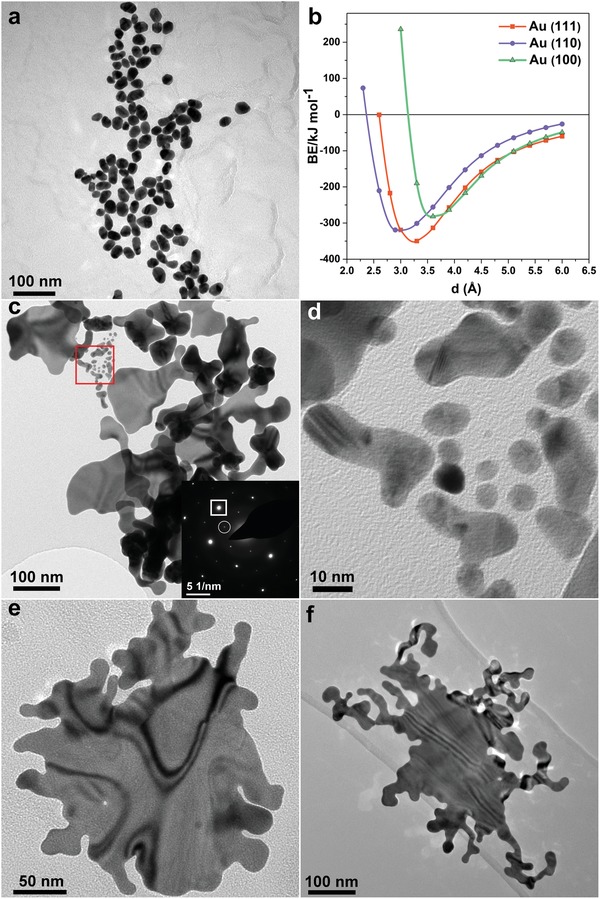
Investigation on the formation mechanism for AuNSWs: product synthesized in the absence of MO with all other reaction parameters unaltered: a) TEM image; b) Binding energies of MO molecule on different Au crystal planes as a function of the deposition height, calculated by using the density functional theory (DFT) at M06‐2X/def2‐SVP level with Grimme's dispersion correction; Characterization of the products collected at different reaction stages: c) TEM image of the product after 2 min of reaction. Inset shows the SAED pattern indexed to the [111] zone axis: strong spots (boxed) indexed as the allowed {220} Bragg reflection (corresponding to a lattice spacing of 0.144 nm); weak spots (circled) indexed as the forbidden 1/3{422} reflections (corresponding to a lattice spacing of 0.250 nm). d) High‐magnification TEM image showing an enlarged view corresponding to the red box in (c). TEM image of the product following e) 10 min and f) 20 min of reaction.

Given the reported simulation work regarding preferred adsorption structures for benzene on metal surfaces,^31^ we have set up a model with MO molecule oriented parallel to the surfaces for calculating the binding energies on different crystallographic facets, using the density functional theory (DFT) at M06‐2X/def2‐SVP level with Grimme's dispersion correction^32^ as a function of the deposition height. Three models (Figure S9, Supporting Information) were employed to present Au (111), Au (110), and Au (100) surfaces with MO molecule oriented parallel to the surfaces. All calculations were done using the program Gaussian.^33^ As shown in Figure 3b, binding energies of MO between Au (111), Au (110), and Au (100) are ≈349.8, 319.2, and 281.6 kJ mol^−1^, respectively. These results suggest that the interfacial interaction between Au (111) surface and MO is the strongest among three surface models. The stronger affinity of MO molecules on the basal (111) planes of Au nanosheets restricts the growth along the [111] direction and contributes to directing the formation of 2D structure. This confinement effect based on the preferential adsorption is similar to that in the carbon monoxide‐confined growth strategy for preparing freestanding ultrathin Pd or Rh nanosheets, whereby the strong adsorption of carbon monoxide on the (111) surface of Pd^9^ or Rh^11^ is believed to be the key factor for prohibiting the isotropic growth and thus producing ultrathin 2D nanostructures.

In addition, we have conducted control experiments of syntheses with lower concentration of MO (0.07 × 10^−3^ m) to elucidate the function of the above‐mentioned 2D molecular stacking of MO in regulating the growth of AuNSWs. The inability of MO to form 2D stacking at low concentration (below 0.20 × 10^−3^ m)^27^, ^28^ allows us to examine whether the preferential adsorption is the only contributing factor. Synthesis with 0.07 × 10^−3^ m MO, whist keeping all the reaction parameters the same with those in the preparation of AuNSWs, generated mostly quasi‐spherical nanoparticles and limited number of small nanoflakes (Figure S10a, Supporting Information). To study whether the failure of growing a 2D gold nanostructure with low concentration (0. 07 × 10^−3^ m) of MO arises from the absence of 2D stacking or insufficient coverage of MO on the (111) surface, we further carried out synthesis with 0. 07 × 10^−3^ m MO, 0. 167 × 10^−3^ m HAuCl_4_ and 33.3 × 10^−3^ m SC for keeping the ratio of MO: HAuCl_4_: SC identical to that in the synthesis of AuNSWs to confer effective surface coverage. The products (Figure S10b,c, Supporting Information) are predominantly the aggregates of nanoflakes and nanoparticles, i.e., the growth of 2D nanocrystal occurred to some extent due to the preferential adsorption of MO, but not well‐confined in a 2D space to produce the final product of Au nanosheets, which implies that 2D planar stacking of MO is also a contributing factor for creating 2D gold sheets. Taken together, MO has dual roles in regulating the formation of <111> oriented AuNSW: the effect of soft template provided by the 2D planar stacking and preferential adsorption on Au (111) for restricting the growth along the [111] direction.

We also characterized the products at different stages during formation using TEM (Figure 3c–f) and UV–vis (Figure S11, Supporting Information). The products collected at 2 min after the addition of SC comprised nanoflakes of varying lateral dimensions (Figure 3c) spanning from several nanometers to about one hundred nanometers. The UV–vis spectrum displayed a wide absorption in the near‐infrared region evidencing the formation of anisotropic nanostructures, in agreement with the TEM observations. Notably, the nanoflakes having lateral dimensions of ≈20–30 nm (Figure 3d) displayed a combination of twins, low‐angle grain boundaries as well as more mis‐oriented boundaries, indicating the coalescence of smaller initially‐formed nanoflakes (of lateral dimension of ≈5 nm). The SAED pattern (inset in Figure 3c) taken from a much larger individual nanoflake with a lateral dimension of ≈100 nm demonstrated a single‐crystal nature with a <111> orientation. These observations are similar to the phenomenon reported in the formation of atomically‐thin PbS^34^ and SnSe nanosheets,^35^ which exhibit coalescence of small crystal nuclei in an orientated attachment mechanism, followed by the transition from a poly‐crystalline mesocrystal to a single‐crystalline structure. Previous work on PdS nanosheets formed via orientated attachment has also demonstrated that, intermediate nanostructures exhibiting low‐angle grain boundaries tend to undergo a surface reconstruction though diffusion and accommodation, resulting in single‐crystalline nanosheets with flat surface that are thinner than the original building blocks.^34^ With increasing reaction time, the lateral dimension of the products increases and the shape assumes a branched fractal structure, with the values of fractal dimension of final products (AuNSWs) indicating the formation via a diffusion‐limited aggregation pathway (as discussed above). Therefore, we propose that the production of AuNSWs includes the following steps: 1) The affinity between Au (III) and aromatic rings^36^, ^37^ leads the gold precursor HAuCl_4_ to be associated with MO molecules, and subsequently reduced by SC to generate primary Au nucleus; 2) Growth of primary nucleus into Au nanoflakes of small lateral size, as confined by MO molecules; 3) Selective oriented attachment of small Au nanoflakes onto the side facets to minimize their high surface energy.^38^, ^39^ The resultant larger‐sized nanoflakes would serve as new nuclei for further 2D attachment of freshly‐produced Au nanoflakes (or Au atoms), following a diffusion‐limited aggregation pathway, gradually evolving into AuNSWs with the observed fractal dimension. An in‐depth comprehension of the formation mechanism, in particular the role of MO, deserves further investigation. Nevertheless, sub‐nanometer thick Au nanosheets can be readily prepared via our MO‐assisted strategy.

Owing to their large surface areas and the ultrathin nature with only two atomic layers, we expect our AuNSWs to possess rich exposed catalytically‐active sites.^3^, ^40^ We evaluated the catalytic performance of AuNSWs, using the model reaction of 4‐nitrophenol (4‐NP) reduction by NaBH_4_, a well‐established benchmark reaction to assess the catalytic activity of various noble metal nanocatalysts.^7^, ^41^ Au nanoparticles (NPs, shown in Figure 3a), synthesized in the absence of MO, were also tested under the same conditions as a control. The reaction can be spectroscopically monitored by measuring the intensity of the characteristic peak at 400 nm corresponding to 4‐NP under an alkaline condition. For the system of 4‐NP and a large excess of NaBH_4_, the 400 nm peak showed negligible change during 2 h (**Figure** **4**a), implying that the reduction of 4‐NP did not occur in the absence of catalysts. In contrast, with the addition of 12 µg AuNSWs or AuNPs, the peak at 400 nm exhibited a gradual decay, accompanied by the appearance of a new peak around 310 nm assigned to the final product of 4‐aminophenol (4‐AP), hence substantiating the catalytic effect of AuNSWs/ AuNPs. The absence of an isosbestic point in Figure 4b,c can be attributed to two factors, including 1) scattering interference with the absorbance measurements resulting from the gas bubbles of H_2_ generated by the decomposition of NaBH_4_ 42 and 2) the formation of multiple intermediates that also absorb in this spectral region,^43^ accompanied by the unambiguous production of 4‐AP. Notably, the reduction reaction was completed in ≈3 min with AuNSWs as the nanocatalyst, whereas ≈30 min with AuNPs. This indicates significantly superior catalytic activity of AuNSWs relative to that of AuNPs. Given that the concentration of NaBH_4_ is in significant excess of 4‐NP, we calculated the reaction rate constants (*k*_app_) using pseudo‐first‐order kinetics^44^ (see the Supporting Information for details), The plots of ln(*C*_t_/*C*_0_) and reaction time showed a linear fit (Figure 4e). The slope, i.e., *k*_app_ was determined to be 1.3 and 0.11 min^−1^ for the reactions using AuNSWs and AuNPs respectively. We also measured the *k*_app_ for the same reaction system using varying amounts of nanocatalyst (AuNSWs or AuNPs, Figure 4f). The calculated *k*_app_ values were proportional to the amount of Au in the reaction system. Thus, according to the equation *k*_1_ = *k*_app_/*M*_Au_, we derived *k*_1_, which reflects the intrinsic catalytic activity and enables a reasonable comparison with previous reported catalysts based on noble metal nanomaterials.^7^, ^44^ The slope (*k*_1_) associated with AuNSWs and AuNPs were calculated to be 1.1 × 10^5^ and 9.2 × 10^3^ min^−1^ g^−1^ respectively, which reveals that the catalytic activity of AuNSWs is 10 times larger than that of AuNPs in this model reaction. Notably, AuNSWs exhibit a superior catalytic activity to most reported noble metal nanocatalysts (Table S1, Supporting Information), and maintain ≈90% of such a high activity after 6 repeated cycles (Figure 4f), demonstrating our AuNSWs can be used as a highly efficient and stable heterogeneous catalyst.

**Figure 4:**
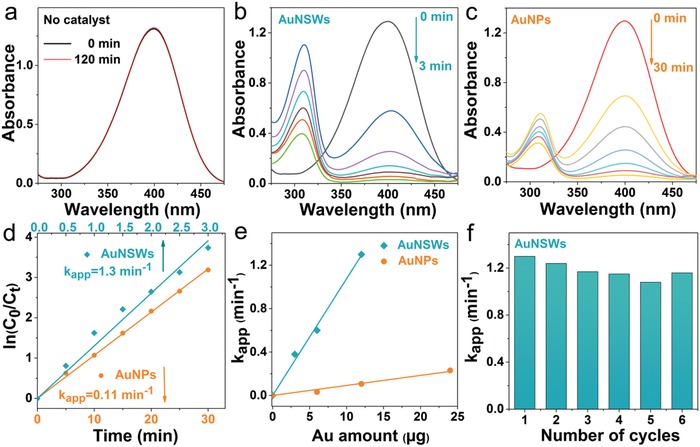
Catalytic performance of AuNSWs in the model reaction of 4‐nitrophenol reduction to 4‐aminophenol: Absorption spectra recorded at different time points in the reaction system: a) in the absence of catalyst; b) in the presence of 12 µg AuNSWs (time interval: 0.5 min); c) in the presence of 12 µg AuNPs (time interval: 5 min); d) Plots of ln(*C*_t_/*C*_0_) as a function of reaction time (amount of AuNSWs/AuNPs: 12 µg); e) plots of the *k*_app_ values versus Au amount; f) *k*_app_ versus the number of successive cycles (12 µg AuNSWs).

Motivated by the excellent catalytic performance of the AuNSWs shown in the model reaction above, we further explored the applicability of AuNSWs as nanoenzymes. Nanomaterials with enzyme‐mimicking catalytic properties, that is, nanoenzymes, have recently emerged as promising artificial enzymes, which exhibit unique catalytic activities, while circumventing the high cost and low stability of natural enzymes. In addition, nanoenzymes have several advantages over molecular artificial enzymes, including diverse functions beyond catalysis and large surface areas facilitating bioconjugation.^45^, ^46^, ^47^, ^48^ Among a variety of nanoenzymes, significant effort has been taken to engineer perooxidase‐mimicking nanoenzymes owing to a broad spectrum of applications, ranging from diagnosis and therapy, to water treatment and antibiofouling coating.^45^, ^47^ We assessed the intrinsic peroxidase‐mimicking capability of AuNSWs with peroxidase substrates 3,3′,5,5′‐tetramethylbenzidine (TMB).^45^, ^48^ As shown in **Figure** **5**a, no obvious color or spectral peak was observed in the system of TMB, TMB + H_2_O_2_ or TMB + AuNSWs, while a blue color, corresponding to the absorbance peaks at 652 and 370 nm, appear in the system of TMB + AuNSWs + H_2_O_2_. The plots of the absorbance at 652 nm with reaction time (inset of Figure 5b) display typical features of enzyme kinetics, that is, a linearity at the initial stage of the reaction, with the reaction rate continuously decreasing before reaching a plateau.^48^, ^49^ These results verify that AuNSWs exhibit a peroxidase‐mimicking activity. Similar to horseradish (HRP) and other nanoenzymes, this catalytic activity is enhanced with increasing AuNSW concentration (Figure 5c), and dependent on the pH as well as temperature (Figure 5d).^45^, ^49^ The optimal pH and temperature were ≈3.5 and 40 °C respectively. Intriguingly, AuNSWs retain 52% of activity at pH 7.0 as compared with that at pH 3.5 and display high catalytic activity in the physiologically‐important pH range of 5.0–7.4, which would be desired for potential biomedical applications under physiological conditions.^49^, ^50^ In order to benchmark the catalytic activity of AuNSWs with HRP, we measured apparent steady‐state kinetic parameters at 37 °C and pH 3.5.^45^, ^48^ Typical Michaelis–Menten curve was obtained in a certain range of TMB or H_2_O_2_ concentrations. The data were fitted following the Michaelis–Menten model (Figures S12 and S13, Supporting Information), to determine Michaelis–Menten constant (*K*_m_) and maximum initial velocity (*V*_max_), with results shown in Table S2 (Supporting Information). *K*_m_ is a parameter indicating the enzyme affinity with substrates, and a low *K*_m_ value represents a strong affinity. The apparent *K*_m_ value of AuNSWs with TMB as the substrate was about 1/3 of that of HRP, revealing that AuNSWs have a higher affinity with TMB than HRP. The apparent *K*_m_ of AuNSWs with H_2_O_2_ as the substrate was remarkably larger than that for HRP, suggesting that a higher H_2_O_2_ concentration was required to reach maximal activity, in accordance with previously reported observations regarding artificial enzyme mimics.^45^, ^49^ Impressively, the *K*_cat_ values of AuNSWs and HRP with H_2_O_2_ substrate are respectively 2.24 × 10^5^and 3.48 × 10^3^ s^−1^,^45^ demonstrating AuNSWs possess a catalytic activity ≈60 times higher than HRP. This large peroxidase‐mimicking activity of AuNSWs enables a highly sensitive colorimetric detection of H_2_O_2_. As can be seen in Figure 5e, the absorbance at 652 nm of the colorimetric sensing systems increases with an increase of H_2_O_2_ concentration from 0.1 to 1000 × 10^−6^ m. Figure 5f depicts a representative H_2_O_2_ dose‐response curve, with the inset showing a linear range between 0.1 and 10 × 10^−6^ m (*y* = 0.02344*x* + 0.00262, *R* ^2^ = 0.999). The limit of detection (LOD) is 0.11 × 10^−6^ m, lower than that of other reported nanoenzymes (Table S3, Supporting Information).

**Figure 5:**
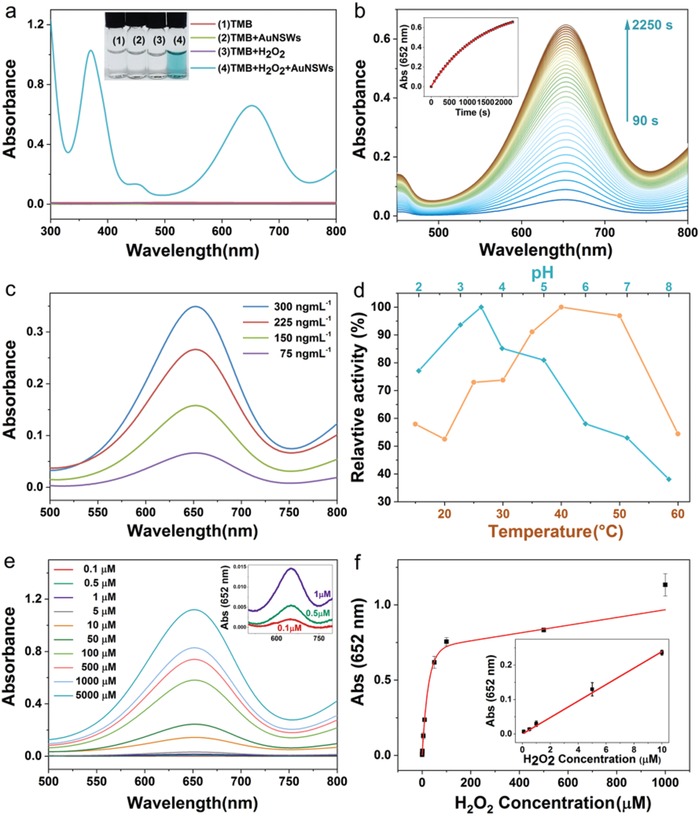
Exploration of the peroxidase‐mimicking function of AuNSWs: a) Digital images and absorption spectra of different TMB‐containing systems: 1) TMB, 2) TMBS + AuNSWs, 3) TMB + H_2_O_2_ and 4) TMB+H_2_O_2_+AuNSWs. b) Absorption spectra recorded at different time points (Time interval: 60 s), in the reaction system of TMB + H_2_O_2_ + AuNSWs, with inset showing the absorbance at 652 nm as a function of reaction time; c) Absorption spectra of the reaction systems of TMB + H_2_O_2_ + AuNSWs, with different concentrations of AuNSWs. d) Effect of temperature or pH value on the peroxidase‐mimicking activity of AuNSWs; e) Absorption spectra of sensing assays using AuNWSs as a peroxidase‐mimicking nanoenzyme, with varying H_2_O_2_ concentrations. f) H_2_O_2_ dose‐response curve, with inset showing the linear calibration plot of H_2_O_2_ concentration. Error bars represent the standard deviation derived from triplicate measurements.

In summary, atomically‐thin 2D AuNSWs have been successfully prepared by a simple aqueous synthesis at an ambient temperature. This synthesis strategy relies on the use of MO molecules to provide confinement during nanocrystal formation. The resultant AuNSWs represent the first free‐standing 2D gold with a sub‐nanometer thickness, and display pronounced catalytic performance toward the reduction of 4‐niotrophenoal as well as the degradation of H_2_O_2_. It is also demonstrated that these sub‐nanometer thick AuNSWs can be readily exploited in the sensing systems based on peroxidase‐mimicking activity, such as for the highly sensitive colorimetric detection of H_2_O_2_. We envisage that the ease of production and unique structural properties of AuNSWs endow them with a variety of potential applications in catalysis, sensors, biomedicine and so on. Our synthesis strategy opens new avenues to the bottom‐up preparation of ultrathin 2D metal nanostructures with enhanced performance and multiple functionalities, which would potentially extend our understanding of the underlying fundamental science and may lead to the unveiling of unprecedented phenomena and properties.

## Experimental Section

Experimental details and any associated references are available in the Supporting Information of this communication. The data presented in this article will be openly available from the University of Leeds data repository https://doi.org/10.5518/464.

## Conflict of Interest

S.Y. and S.D.E. have filed a patent (Application number: 1818923.3) relating to 2D metal nanomaterials.

## Supporting information

Supplementary
