# Supplementary material for: Sub‐Nanometer Thick Gold Nanosheets as Highly Efficient Catalysts
Source: Adv Sci (Weinh). 2019 Aug 6;6(21):1900911. doi: 10.1002/advs.201900911 (PMC6839621; doi:10.1002/advs.201900911)
Supplement: Supplementary file 1 — Supplementary [file ADVS-6-1900911-s001.pdf]

Copyright WILEY-VCH Verlag GmbH & Co. KGaA, 69469 Weinheim, Germany, 2019.

ADVANCED SCIENCE
Open Access

Supporting Information

for *Adv. Sci.,* DOI: 10.1002/advs.201900911

Sub-Nanometer Thick Gold Nanosheets as Highly Efficient Catalysts

*Sunjie Ye, Andy P. Brown, Ashley C. Stammers, Neil H. Thomson, Jin Wen, Lucien Roach, Richard J. Bushby, Patricia Louise Coletta, Kevin Critchley, Simon D. Connell, Alexander F. Markham, Rik Brydson, and Stephen D. Evans**

Copyright WILEY-VCH Verlag GmbH & Co. KGaA, 69469 Weinheim, Germany, 2019.

Supporting Information

*Sunjie Ye, Andy P. Brown, Ashley C. Stammers, Neil H. Thomson, Jin Wen, Lucien Roach, Richard J. Bushby, P. Louise Coletta, Kevin Critchley, Simon D. Connell, Alexander F. Markham, Rik Brydson, Stephen D. Evans**

Dr. S. Ye, Dr. A. C. Stammers, L. Roach, Prof. R. J. Bushby, Dr. K. Critchley, Prof. S. D. Connell, Prof. S. D. Evans
School of Physics and Astronomy
University of Leeds
Leeds, LS2 9JT, UK
E-mail: S.D.Evans@leeds.ac.uk
Dr. S. Ye, Dr. P. L. Coletta, Prof. A.F. Markham,
Leeds Institute of Medical Research, St James's University Hospital,
University of Leeds,
Leeds, LS9 7TF, UK
Prof. A. P. Brown, Prof. R. Brydson
School of Chemical and Process Engineering,
University of Leeds,
Leeds, LS2 9JT, U.K
Dr. N. H. Thomson
Division of Oral Biology, School of Dentistry,
University of Leeds,
Leeds, LS9 7TF, UK
Dr. Jin Wen
Institute of Organic Chemistry and Biochemistry, AS CR, 166 10 Praha 6, Czech Republic

Methods

Materials:

Gold (III) chloride trihydrate (520918), 4-nitrophenol (Fluka-73560), 3,3´,5,5´-tetramethylbenzidine (TMB, 860336) and 2,2"-Bipyridyl (D216305) were purchased from Sigma-Aldrich. Trisodium citrate, anhydrous (45556), Methyl Orange (17874), and sodium acetate trihydrate (A16230) were purchased from Alfa Aesar. Hydrochloric acid (37%, UN1789), and nitric acid (70%, UN2031), sodium borohydride ($NaBH_4$, 10599010), hydrogen peroxide (H_2O_2, H/1750/15), potassium chloride (P/4240/53) and acetic acid (A/0360/PB08) were purchased from Fisher Scientific. Phosphate-Buffered Saline (PBS)

tablets (18912-014) were purchased from life technologies™. All chemicals were used without further purification. Milli-Q water (18.2 MΩ·cm at 25°C) was used in all the experiments.

Characterizations:

UV-Vis spectra: UV-Vis spectra were recorded with a UV-Vis-NIR spectrophotometer (Perkin Elmer Lambda 19 or Agilent Technologies Cary 5000).

AFM imaging and analysis: AFM height measurements were used to determine the thickness of the AuNSWs. The samples were imaged on a Dimension FastScan Bio AFM (Bruker, Billerica MA) using tapping mode at room temperature in air with FastScan-A cantilever probes (Bruker, Camarillo CA). Accurate calibration of the Z-piezo was confirmed by measuring the depth of pits on HF-etched muscovite mica (Figure S1). The terraces created by HF-etching are 1.00nm high, which represents half the c-axis spacing of the monoclinic unit cell.[1] HF mica was prepared by incubating freshly cleaved mica sheets in 40% HF for four hours. The HF was neutralised in an excess of sodium bicarbonate and Milli Q water before imaging. Prior to thickness measurement by AFM, samples were subject to copious washing by Milli Q water (centrifugation and re-dispersion) to remove possible surface ligands. 2 μl of AuNSW samples was deposited onto freshly cleaved muscovite mica and left at room temperature, allowing the water to evaporate. Images were typically acquired at scan sizes of 1 to 5 μm with a resolution of 2048 x 2048 pixels at 10.5 Hz scan rate. The FastScan A cantilever (Bruker) was tuned to 5% below resonance to operate in repulsive mode tapping with a typical resonant frequency of 1.4 MHz. The amplitude setpoint was reduced such that the tapping mode phase angle was reduced to around 1 degree contrast, representing a minimal difference in amplitude response between the sample and substrate, hence eliminating height errors due to differences in tip-sample interaction. Analysis of sample heights were performed in Bruker Nanoscope Analysis v1.9. Height measurements of the flakes by AFM were performed by fitting Gaussian peaks to a depth histogram. Due to 0.1-

0.2 nm amplitude noise the heights measured from individual scan lines or averaged scan lines are prone to error. These are both random noise, in the form of low frequency z-scan instability, high frequency gain noise, and systematic noise due to scanner ringing on turnaround causing sinusoidal oscillation at this reasonable high scan rate, and z-overshoot on steps in the scan direction. Using higher order plane fits on individual flakes cropped from pre-flattened images, together with median filtering, noise could be averaged, and the height histogram peaks could be clearly discriminated.

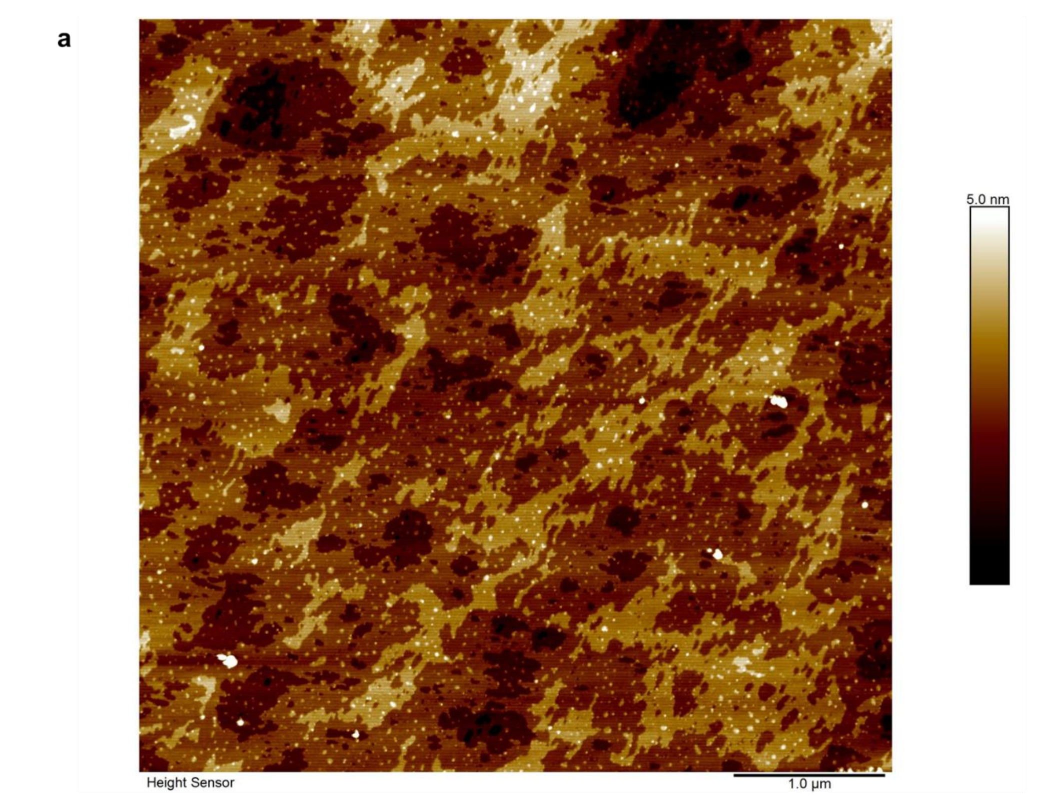

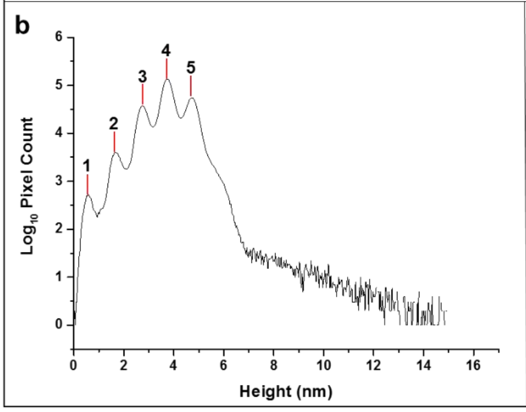

c

Peak no.	Height (nm)	Difference (nm)
1	0.584	
2	1.694	1.109
3	2.746	1.051
4	3.739	0.993
5	4.731	0.993

Figure S1: Calibration of the AFM Z-piezo using HF-etched mica: a) Tapping mode AFM image of HF-etched muscovite mica. Image size: 5 x 5 µm Pixels: 2048 x 2048 b) Height distribution of the AFM image data showing five terrace levels (total pixel count: 2048^2). c) Summary of the heights of the five terraces and the differences between them, confirming accuracy of measurement to mica step height to within 4% (Mean height: 1.04 ± 0.05 nm SD).

XRD pattern: XRD patterns were obtained using a Bruker D8 X-ray diffractometer with Cu Kα source and a Vantec-1 detector. A continuous scan over a 2θ range from 20° to 90° was performed with an acquisition time of 5 hours, at a step size of 0.016°. The sample was prepared by depositing and drying slurries directly on low-background Si sample holders.

Atomic absorption spectrometer (AAS) measurement:The content of gold nanostructures (AuNSWs and AuNPs) in solution was measured using an atomic absorption spectrometer (Varian 240fs).

Dynamic light scattering (DLS): The hydrodynamic diameters of AuNPs (synthesized in the absence of MO) were measured by (DLS) using Zetasizer Nano ZSP (Malvern Instruments) at 25°C, in a detection angle of 13° forward.

TEM imaging and SAED: TEM images and SAED patterns were collected using a range of microscopes at the University of Leeds: a Tecnai G2 Spirit TWIN/BioTWIN at an acceleration voltage of 120 kV, a Tecnai F20 TEM/STEM operated at an accelerating voltage of 200 kV, equipped with a field emission gun using an extraction voltage of 4.5 kV, an Oxford Instruments 80 mm^2 SD detector running Aztec software, and a Gatan Orius CCD camera running Digital Micrograph software and a FEI Titan3 Themis G2 S/TEM operated at 300 kV equipped with a monochromator, FEI SuperX EDX detectors, a Gatan Quantum ER 965 imaging filter and a Gatan OneView CCD camera running GMS 3.1.

For preparing TEM samples, 5 µL nanoparticle dispersion (in Milli-Q) was dropped onto a carbon-coated copper grid (Agar Scientific Ltd) and dried at room temperature naturally.

Reaction Preparation:

Vials were cleaned with aqua regia (nitric acid and hydrochloric acid in a volume ratio of 1:3), thoroughly rinsed with Milli-Q water and dried in an 80 °C oven before use. Once dry, the flasks were allowed to cool to room temperature before any reactants were added.

Synthesis of Au Nanoseaweed:

Gold chloride ($HAuCl_4$) aqueous solution (1 mL, 5 mM) and freshly prepared sodium citrate (SC) aqueous solution (0.5 mL, 100 mM) were sequentially added into 4 mL aqueous solution of Methyl Orange (MO, 0.21 mM) at 20°C. The resultant reaction solution was kept undisturbed at this temperature for 12 h (Figure S2 shows the digital images of the reaction solution at different time points). The reaction products were collected by centrifugation (1000g*10min), and then washed several times with Milli-Q water until the supernatant was colourless. The pellet was then redispersed in Milli-Q water.

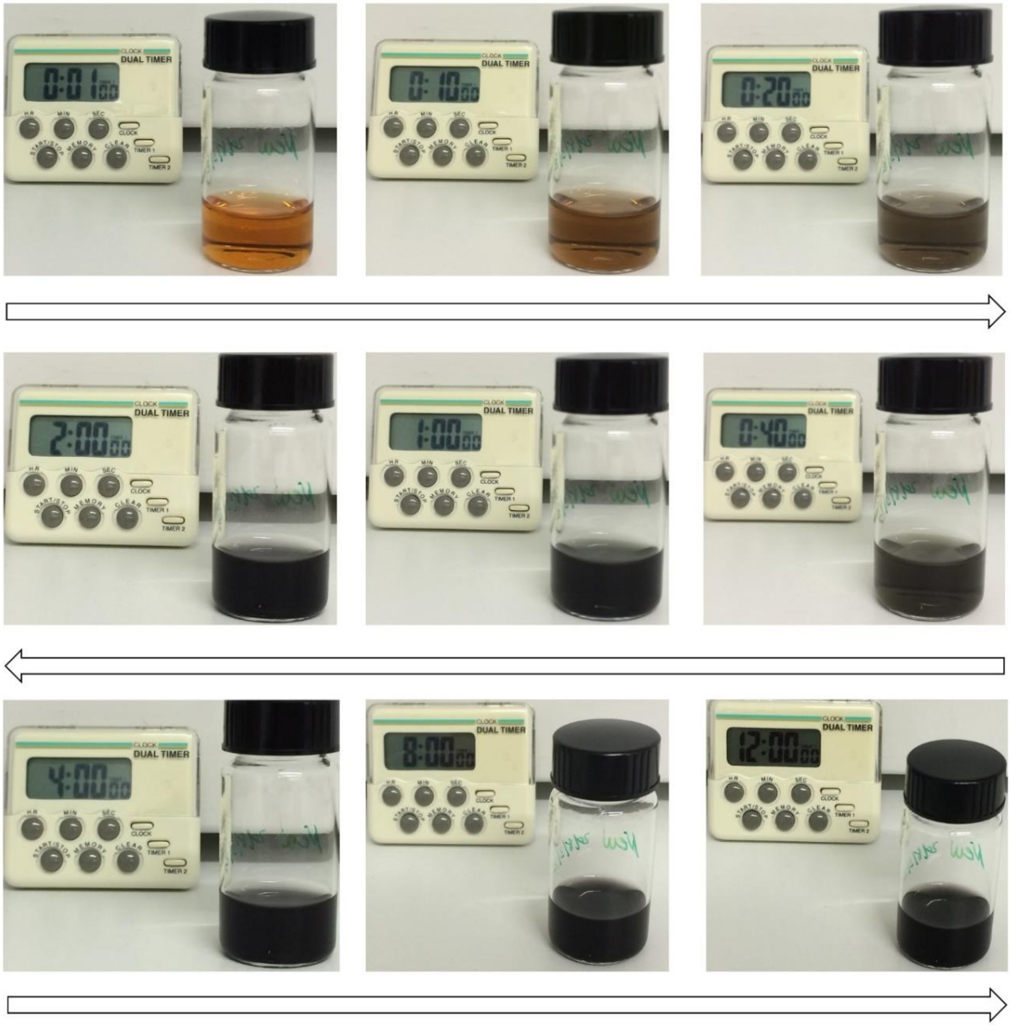

Figure S2 Digital images of the reaction solution at different time points during the reaction.

Synthesis of gold nanostructures in the absence of MO

$HAuCl_4$ aqueous solution (1 mL, 5 mM) and SC aqueous solution (freshly prepared, 0.5 mL, 100 mM) were sequentially added into 4 mL Milli-Q water at 20°C. The resultant reaction solution was kept undisturbed at this temperature for 12 h. The reaction products were collected by centrifugation (1000 g*10 min), and then washed thrice with Milli-Q water. The pellet was then redispersed in Milli-Q water.

Synthesis of gold nanostructures with 0. 07 MO

$HAuCl_4$ aqueous solution (1 mL, 5mM or 1.67 mM) and SC aqueous solution (freshly prepared, 0.5 mL, 100 mM or 33 mM) were sequentially added into 4 mL MO aqueous solution (0.07 mM) at 20°C. The resultant reaction solution was kept undisturbed at this temperature for 12 h. The reaction products were collected by centrifugation (1000 g*10 min), and then washed several times with Milli-Q water until the supernatant is colourless. The pellet was then redispersed in Milli-Q water.

Synthesis with 2,2'-Bipyridine

$HAuCl_4$ aqueous solution (1 mL, 5 mM) and SC aqueous solution (freshly prepared, 0.5 mL, 100 mM) were sequentially added into aqueous solution (0. 21 mM) of 2,2'-Bipyridine at 20°C. The resultant reaction solution was kept undisturbed at this temperature for 12 h. The reaction products formed a precipitation at the bottom of the vial. After the removal of the supernatant, the products were redispersed in Milli-Q water, washed by centrifugation (1000 g*8 min) for several times and then redispersed in Milli-Q water.

Catalysis test using the model reaction of 4-nitrophenol (4-NP) reduction by $NaBH_4$

The freshly-prepared 10 μl of 4-NP aqueous solution (15 mM) and 980 μl of $NaBH_4$ aqueous solution (20 mM), 10 μl of AuNSWs (or AuNPs) dispersions (1200 $\mu g ml^{-1}$, 600 $\mu g ml^{-1}$ or 300 $\mu g ml^{-1}$ for AuNSWs; 2400 $\mu g ml^{-1}$, 1200 $\mu g ml^{-1}$ or 600 $\mu g ml^{-1}$ for AuNPs) were added sequentially into a cuvette at 20 °C and well mixed with a pipette. The kinetic process of the reduction reaction was monitored by absorbance at 400 nm as a function of time. For accuracy, the extinction caused by AuNSWs (or AuNPs) was subtracted from the spectroscopic measurements. Given that the concentration of $NaBH_4$ is in significant excess of 4-nitrophenol, we calculated the reaction rate constants (k_{app}) using pseudo-first-order kinetics (Eqn 1), the apparent rate constant (k_{app}) can be determined by calculating the slope of a linear plot of $\ln(C_0/C_t)$ versus reaction time (t), where C_0 and C_t are the concentrations of 4-NP at time 0 and t, respectively, and C_0/C_t equals to A_0/A_t, where A_0 and A_t are the peak absorbance of 4-NP at time 0 and t, respectively.

$$-\mathrm{d}C_t / \mathrm{d}_t = k_{\mathrm{app}} C_t$$
$$\ln(C_t) = -k_{\mathrm{app}} t + \ln(C_0) \quad (1)$$
$$k_{\mathrm{app}} t = \ln(C_0 / C_t)$$

To evaluate the stability of AuNSWs as nanocatalysts, we tracked the variations in the catalytic activity by repeated addition of new 4-NP.[3] After each cycle of reaction proceeded for 3 mins, 10 μl of 4-NP aqueous solution (20 mM) was added to the reaction system.

Assessment of Peroxidase-mimicking Activity

Tests were performed following standard protocols for determining catalytic activity and kinetics of peroxidase-mimicking nanozymes,[4] using TMB as a colorimetric substrate (absorption coefficient at 652 nm, i.e. $\varepsilon_{652\ \mathrm{nm}}$ = 39,000 M^{-1} cm^{-1}). In a typical test, 20 μL of AuNSW dispersion (final concentration: 0.3 μg/mL), 10 μL TMB solution (final concentration: 0.1 mM), 20 μL H_2O_2 (final concentration: 30 mM) were successively added into acetate buffer (pH 3.5). The absorption spectra were collected on an Agilent Technologies Cary 5000 UV-Vis-NIR spectrophotometer coupled with a Cary temperature controller, using a 1 cm path length quartz cuvette. The absorbance change at 652 nm was monitored for kinetic study. The extinction caused by the AuNSWs was subtracted from spectroscopic measurements. The pH effect on the nanoenzyme activity was evaluated using the above assay, with different buffers: hydrochloric acid-potassium chloride buffer (pH 2), acetate buffer (pH 3.0-5.0) and phosphate buffer (pH 6.0-8.0). The maximal activity in the test was defined as 100% relative activity. The steady-state kinetic analysis of AuNSWs with TMB and H_2O_2 as the substrates was conducted by altering the concentrations of TMB at a fixed H_2O_2 initial concentration and vice versa at a fixed TMB initial concentration. The apparent kinetic parameters were calculated based on Michaelis-Menten function: $\nu=V_{max}[S]/(K_m+[S])$, where ν is the initial velocity, V_{max} is the maximal reaction velocity, [S] is the concentration of substrate and K_m is the Michaelis constant.

Colorimetric detection of H_2O_2

AuNSWs were employed to construct a colorimetric sensing assay for H_2O_2 detection. In each test, 40 µL of AuNSW dispersion (final concentration: 1.5 µg/mL), 20 µL TMB solution (final concentration: 1 mM), 40 µL H_2O_2 (final concentration: 0.1 µM, 0.5 µM, 1 µM, 5 µM, 10 µM, 50 µM, 100 µM, 500 µM, 1000 µM, or 5000 µM) were successively added into 1.9 mL acetate buffer (pH 3.5). The absorption spectra were measured after 30 mins of incubation at 37°C.

Results and discussion

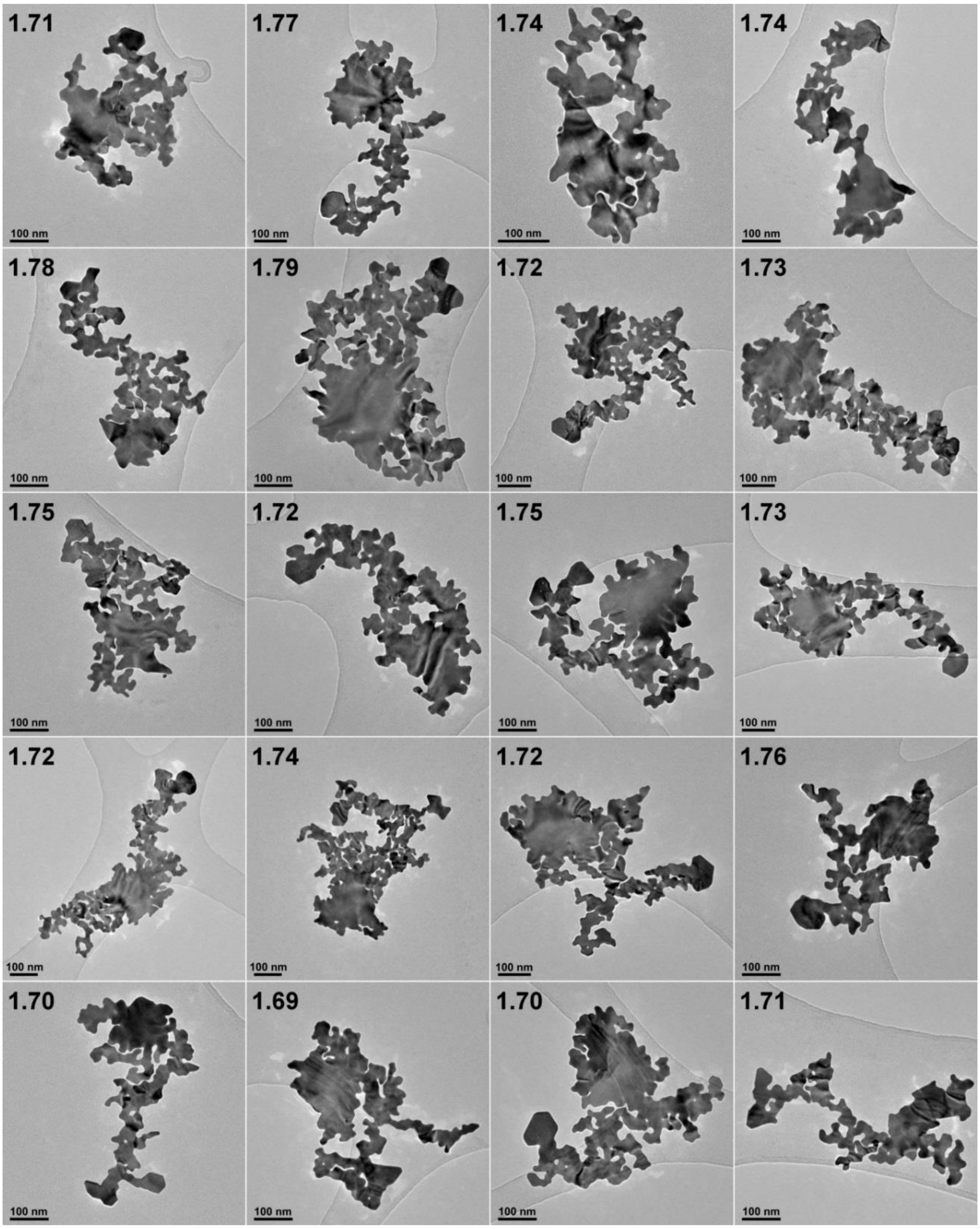

Figure S3 Calculation of Fractal dimension of Au NSWs: Bright field TEM images of non-overlapping individual Au nanoseaweeds. The number inset represents the calculated fractal dimension of the corresponding Au nanoseaweed. The calculation was conducted using the software FDC http://paulbourke.net/fractals/fracdim/. In the calculation, the contrast of the image was adjusted, such that the algorithm correctly identifies the whole shape of individual Au nanoseaweed.

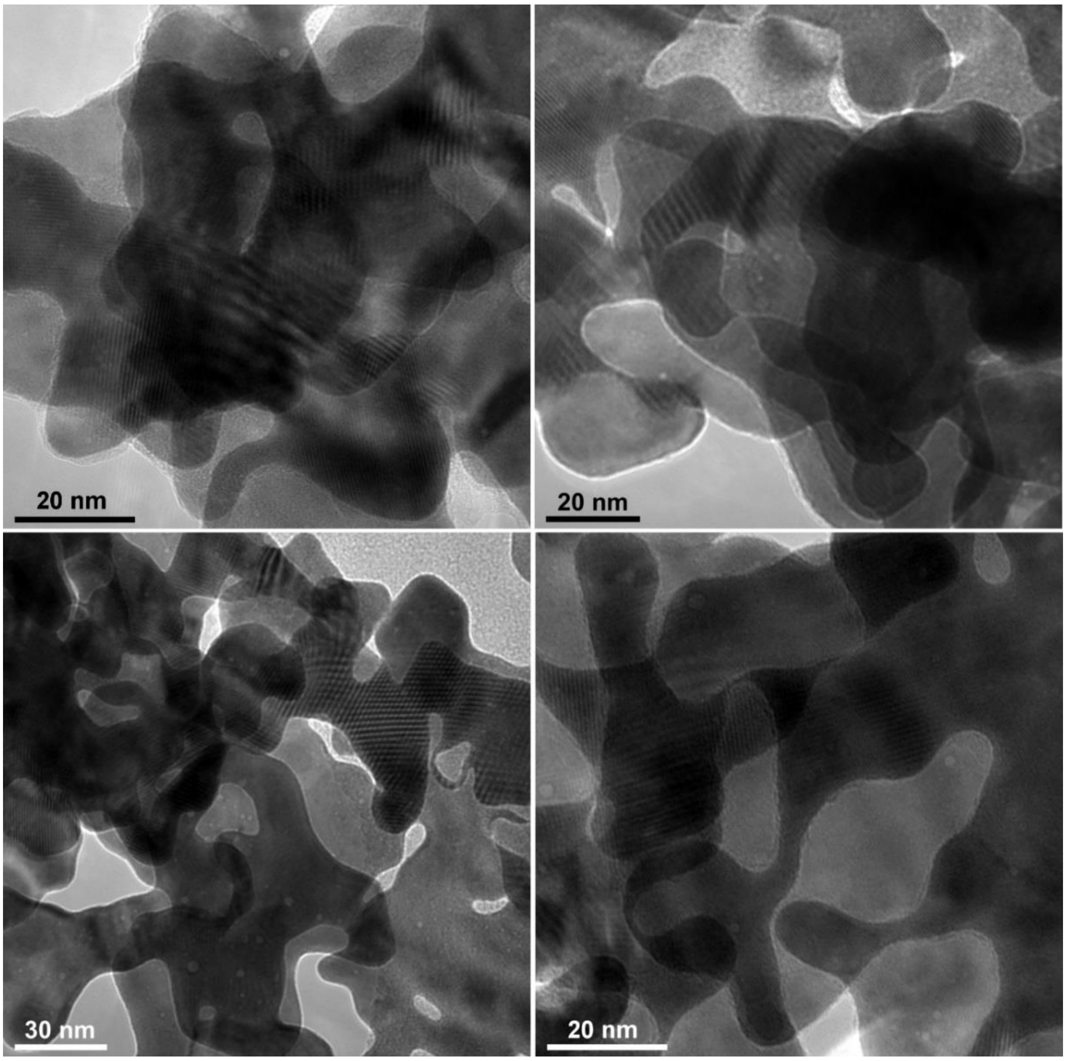

Figure S4 Bright field TEM images showing the translucent appearance, folded edges and wrinkles of AuNSWs, indicating their ultrathin nature.

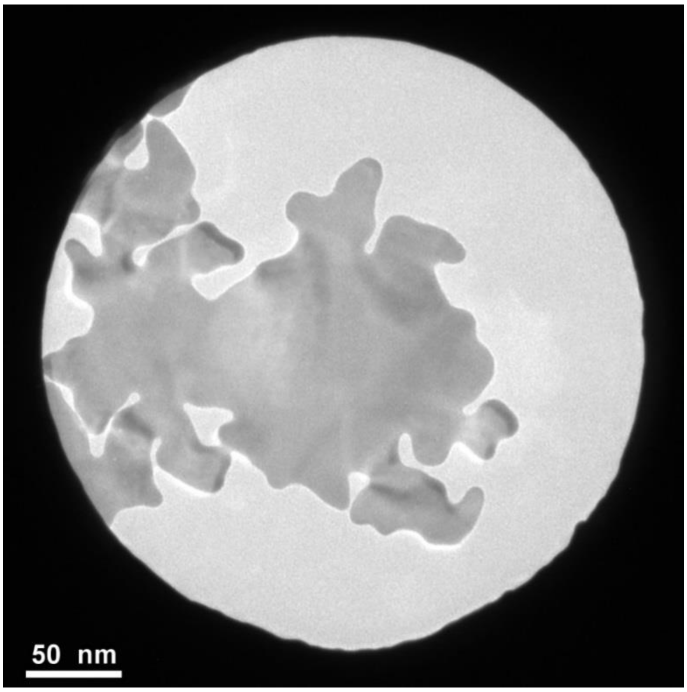

Figure S5 TEM image showing the region corresponding to SAED in Figure 2b

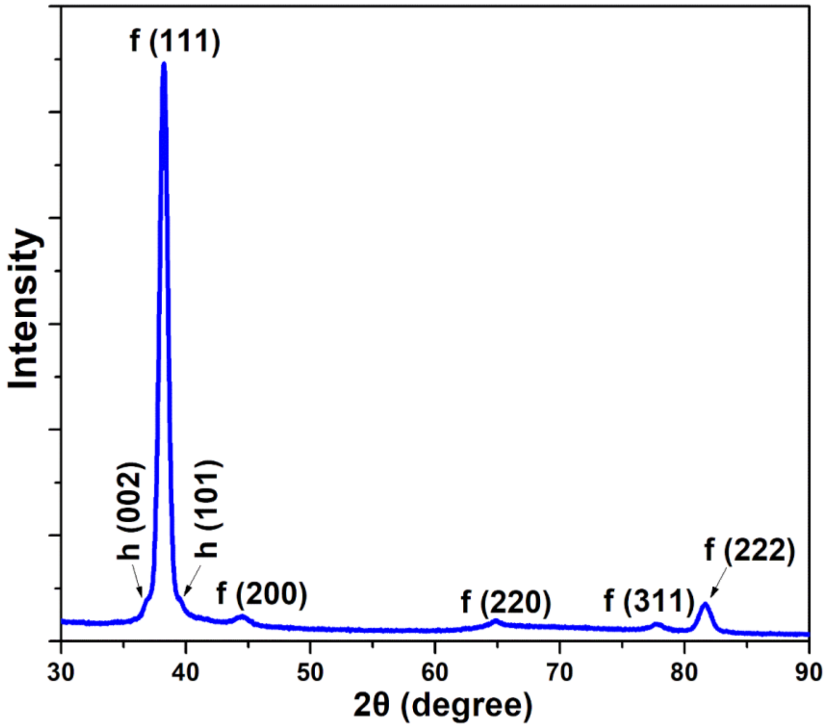

Figure S6 Indexed XRD pattern of AuNSWs over a 2θ range from 30° to 90°.

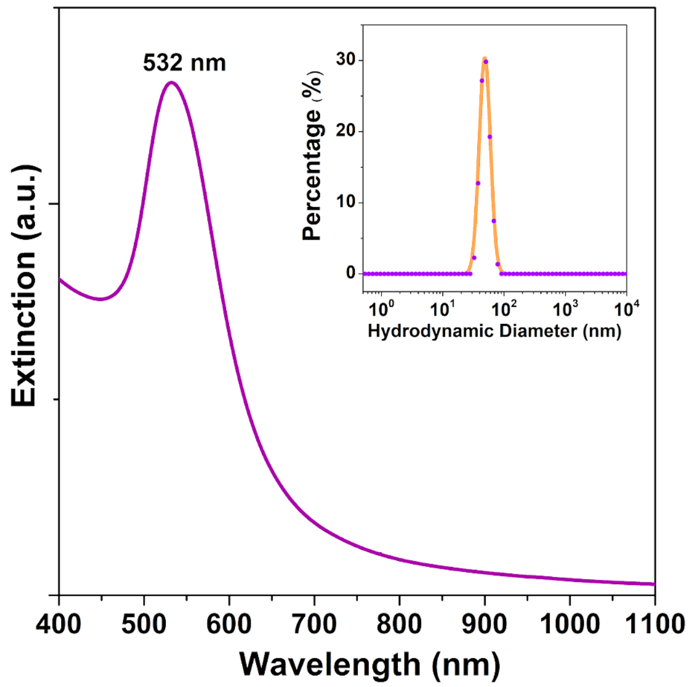

Figure S7 UV-Vis spectrum with inset showing the DLS size distribution

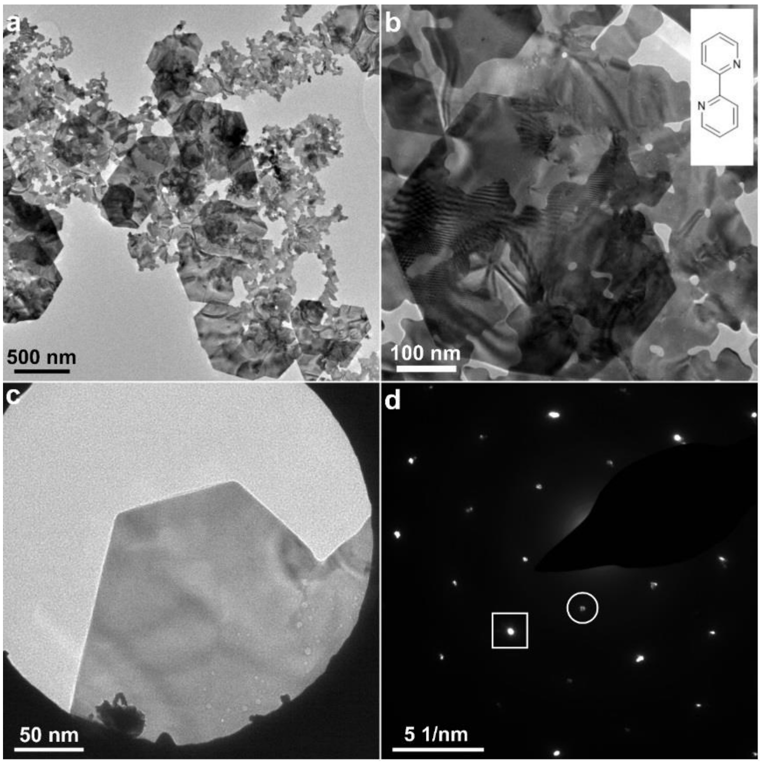

Figure S8 Au nanostructures synthesized using 2,2'-Bipyridine: a-b, Bright field TEM images at different magnifications, demonstrating the high-yield production of 2D gold nanostructures; Inset shows the molecular structure of 2,2'-Bipyridine. c, TEM image showing

the region corresponding to SAED pattern in d; d, SAED pattern down the <111> zone axis: strong spots (boxed) indexed as the allowed {220} Bragg reflection (corresponding to a lattice spacing of 0.144 nm), weak spots (circled) indexed as the forbidden 1/3{422} reflections (corresponding to a lattice spacing of 0.250 nm).

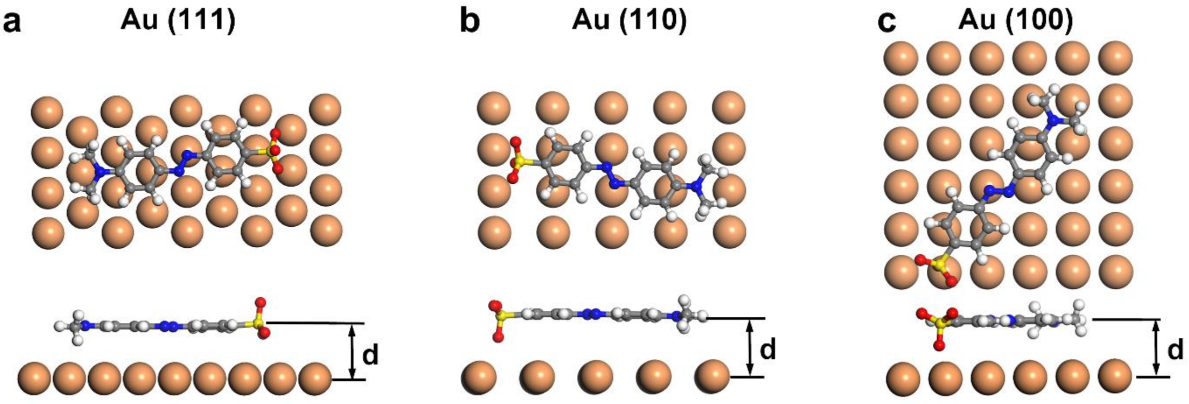

Figure S9 Top and side views of three Au cluster models for a, Au (111), b, Au (110), and c, Au (100) surfaces. The adsorption height, d is defined as the distance between Au surfaces and the plane of the methyl orange molecule.

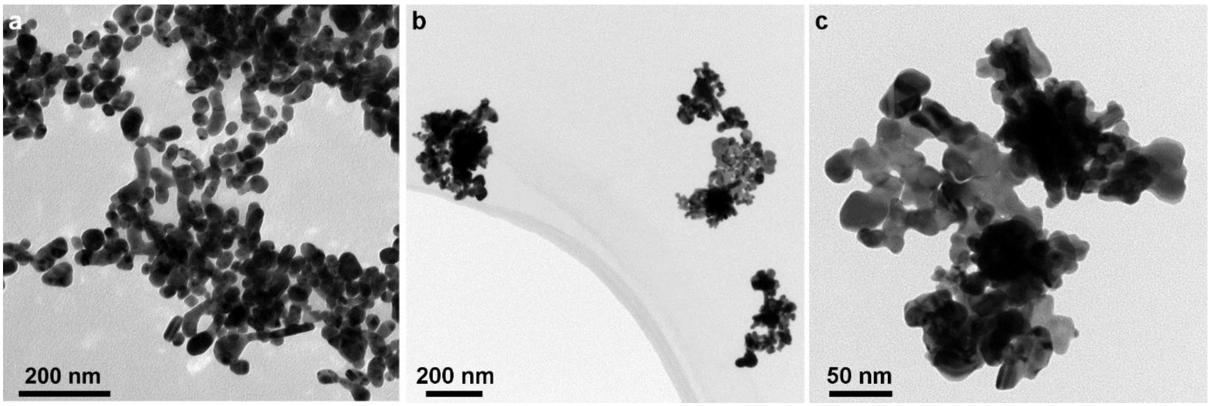

Figure S10 a, Au nanostructures synthesized with 0. 07 mM MO, whist keeping all the reaction parameters constant with those in the preparation of AuNSWs; b, c, TEM images (at different magnifications) of Au nanostructures synthesized with 0. 07 mM MO, 0. 167 mM HAuCl4 and 33.3 mM SC for keeping the ratio of MO: HAuCl4: SC identical with that in the synthesis of AuNSWs.

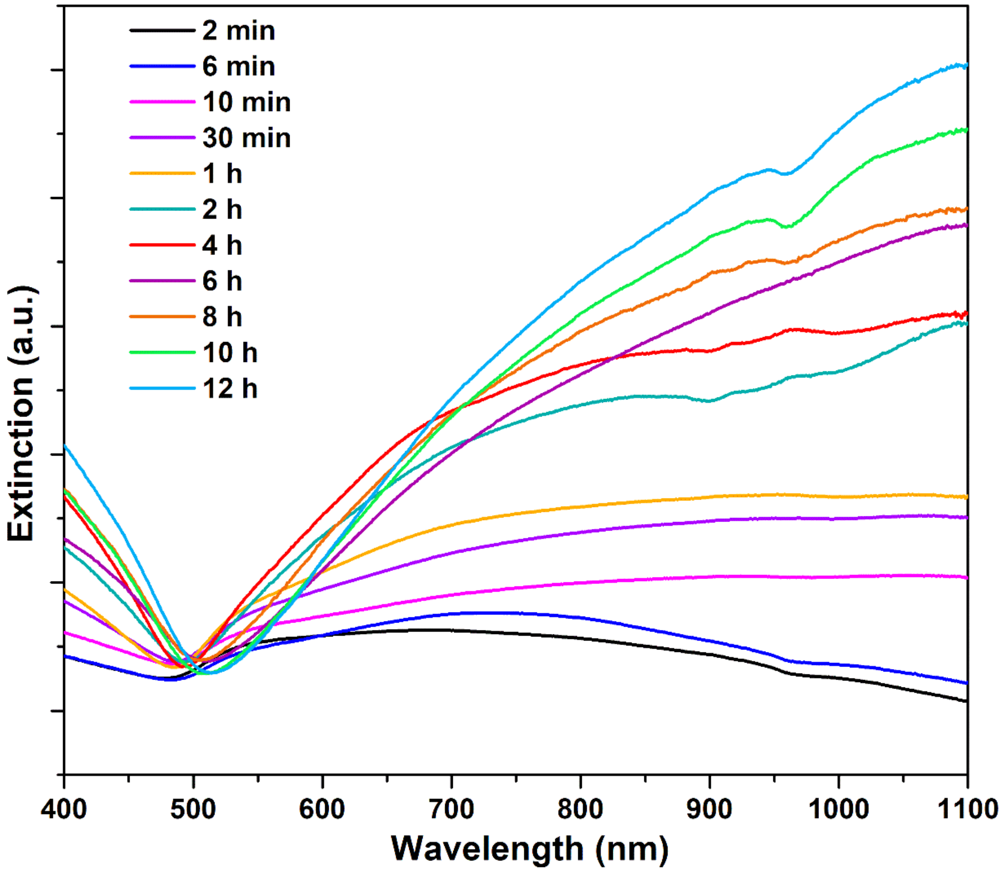

Figure S11 UV-Vis spectra of the product collected at the different stages during the formation of AuNSWs.

Table S1. Comparison of catalytic activities of noble metal-based catalysts using the reduction of 4-nitrophenol by $NaBH_4$ as a model reaction.

Noble metal-based catalysts	Metal amount	Rate constant (k)	Rate constant per unit mass	Reference
DMF-stabilized Au nanoclusters	0.1 μg	0.18 min^{-1}	1.8×10^6 min^{-1} gAu^{-1}	5
Pd@Ru Nanosheets	1.6 μg	0.22 min^{-1}	1.4×10^5 min^{-1} gAu^{-1}	6
AuNSWs	12 μg	1.3 min^{-1}	1.1×10^5 min^{-1} gAu^{-1}	This work
Pd nanosheets	1.6 μg	0.16 min^{-1}	1.0×10^5 min^{-1} $gmetal^{-1}$	6
Au Nanoparticles (14.1 nm in diameter)	5.88 μg	0.53 min^{-1}	9.0×10^4 min^{-1} gAu^{-1}	7
Matryoshka-caged gold nanorods (Hexa-)	NA	NA	3.7×10^4 min^{-1} gAu^{-1}	8
Au@SiO_2	315.2 μg	0.84 min^{-1}	2.7×10^4 min^{-1} gAu^{-1}	9
2D nanosheet of mixed-ligand Ni(II) MOF	16 μg	0.40 min^{-1}	2.5×10^4 min^{-1} gAu^{-1}	10
Au nanostars (8-10 branches)	15.2 μg	0.22 min^{-1}	1.5×10^4 min^{-1} gAu^{-1}	11
AuNPs	12 μg	0.11 min^{-1}	9.2×10^3 min^{-1} gAu^{-1}	This work
Thio-stabilized Au_{25} clusters	94.4 μg	0.51min^{-1}	5.4×10^3 min^{-1} gAu^{-1}	12

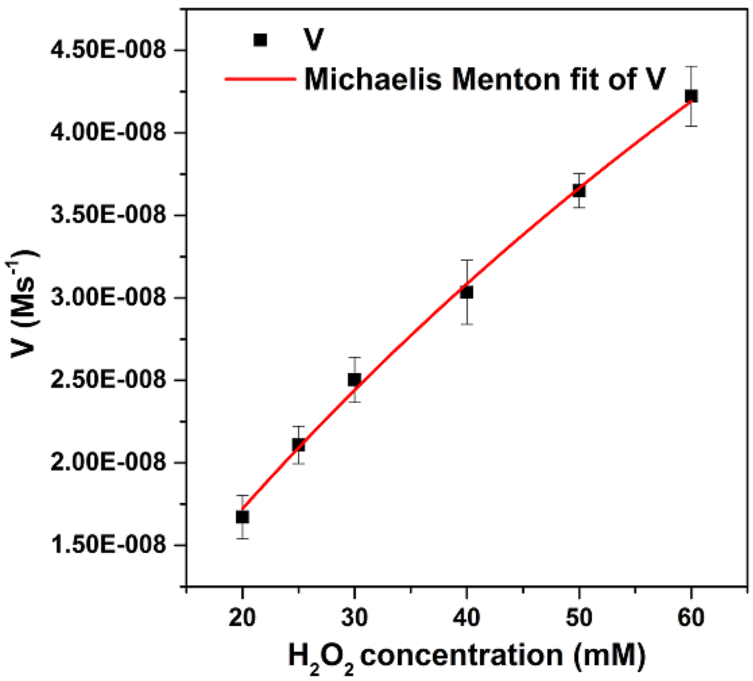

Figure S12 Michaelis–Menten curves using AuNSWs (0.3 μg/mL) as peroxidase mimicking enzymes at 37°C, pH=3.5 (Substrate: H_2O_2, TMB concentration: 0.8 mM). Error bars represent the standard deviation derived from triplicate measurements.

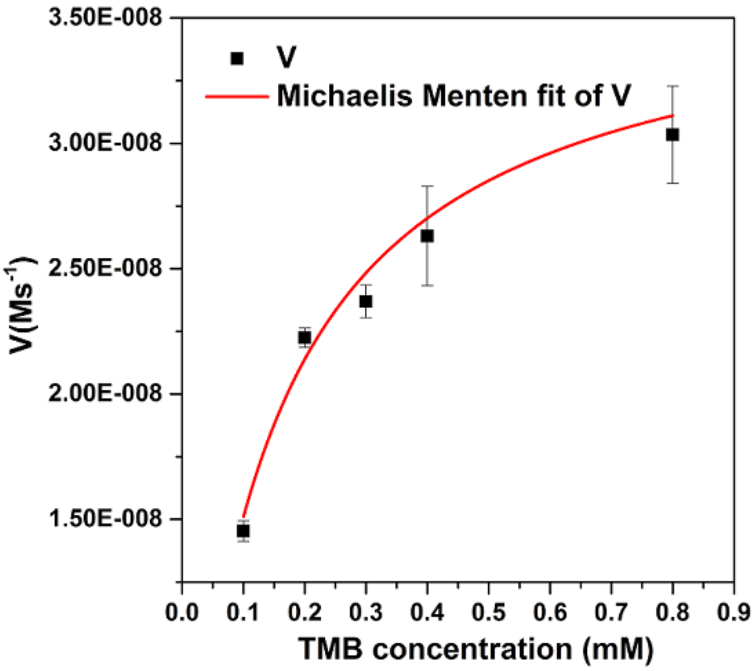

Figure S13 Michaelis–Menten curves for AuNSWs (0.3 μg/mL) as peroxidase mimicking enzymes at 37°C, pH=3.5 (Substrate: TMB, H_2O_2 concentration: 40 mM). Error bars represent the standard deviation derived from triplicate measurements.

Table S2 Comparison of the kinetic parameters of AuNSWs and HRP. [E] is the enzyme (AuNSW) concentration, K_m is the Michaelis constant, V_{max} is the maximal reaction velocity and K_{cat} is the catalytic constant, where $K_{cat}=V_{max}/[E]$.

	**[E](M)**	**Substrate**	**K_m (mM)**	**V_{max} (Ms^{-1})**	**K_{cat}(s^{-1})**
AuNSWs	6.6×10^{-13}	TMB	0.14	3.7×10^{-8}	5.61×10^{4}
HRP [13]	2.5×10^{-11}	TMB	0.434	10×10^{-8}	4×10^{3}
AuNSWs	6.6×10^{-13}	H_2O_2	152	14.8×10^{-8}	2.24×10^{5}
HRP [13]	2.5×10^{-11}	H_2O_2	3.70	8.71×10^{-8}	3.48×10^{3}

Note: For calculating [E] of AuNSWs, the mass content in solution was determined using AAS; Average area was determined to be 85134 nm^2, by averaging the areas of 20 non-overlapping individual AuNSWs (shown in Figure S3) measured using Image J software; Average thickness was determined to be 0.47 nm using AFM.

Table S3 Comparison of LOD and linear range of AuNSWs with other peroxidase-mimicking nanoenzyme for colorimetric detection of H_2O_2

**Nanoenzyme**	**LOD (µM)**	**Linear range**	**Reference**
AuNSWs	0.11	0.1 µM-10 µM	This work
SDS-MoS_2	0.32	2 µM-100 µM	14
MoS_2-$Pt_{74}Ag_{26}$	0.4	1 µM-50 µM	15
$MoSe_2$ nanosheets	0.41	0.01 mM-0.16 mM	16
Chitosan-Au NPs	0.6	1 µM-5 mM	17
MoS_2@$MgFe_2O_4$	1.0	2.5 µM-300 µM	18
Au@Ag nanorods	6	0.01mM-10 mM	19
Prussian Blue@FePt	8.27	20 µM-800 µM	20
Porphyrin-ZnS	15.8	0.01mM-0.06 mM	21

References

[1] N. H. Thomson, H. K. Christenson, H. K. *Surf. Sci. Rep.* **2016**, *17 (2)* 367.

[2] W. B. Xin, J. Severino J, I.M. De Rosa, D. Yu, J. McKay, P. Y. Ye, X. Q. Yin, J. M. Yang, L. Carlson, S. Kodambaka, *Nano Lett*. **2018**, *18(3)*: 1875.

[3] T. Yu, J. Zeng, B. Lim and Y. Xia, *Adv. Mater.* **2010**, *22*, 5188.

[4] B. Jiang, D. M. Duan, L. Z. Gao, M. J. Zhou, K. L. Fan, Y. Tang, J. Q. Xi, Y. H. Bi, Z. Tong, G. F. Gao, N. Xie, A. Tango, G. H. Nie, M. M. Liang, X. Y. Yan, *Nat. Protoc.***2018**, *13*, 1506.

[5] H. Yamamoto, H. Yano, H. Kouchi, Y. Obora, R. Arakawa, H. Kawasaki, *Nanoscale* **2012**, *4*, 4148.

[6] Z. C. Zhang, Y. Liu, B. Chen, Y. Gong, L. Gu, Z. X. Fan, N. L. Yang, Z. C. Lai, Y. Chen, J. Wang, Y. Huang, M. Sindoro, W. X. Niu, B. Li, Y. Zong, Y. H. Yang, X. Huang, F. W. Huo, W. Huang, H. Zhang, *Adv. Mater.* **2016**, *28*, 10282.

[7] P. Suchomel, L. Kvitek, R. Prucek, A. Panacek, A. Halder, S. Vajda, R. Zboril, *Sci. Rep.* **2018**, *8*, 4589.

[8] W. Xiong, D. Sikdar, L. W. Yap, P. Z. Guo, M. Premaratne, X. Y. Li, W. L. Cheng, *Nano Res.* **2016**, *9*, 415.

[9] J. Lee, J. C. Park, H. Song, *Adv. Mater.* **2008**, *20*, 1523.

[10] R. Yan, Y. Zhao, H. Yang, X.-J. Kang, C. Wang, L.-L. Wen, Z.-D. Lu, *Adv. Funct. Mater.* **2018**, *28*, 1802021.

[11] P. Ndokoye, Q. D. Zhao, X. Y. Li, T. T. Li, M. O. Tade, S. B. Wang, *J. Colloid Interface Sci.* **2016**, *477*, 1.

[12] A. Shivhare, S. J. Ambrose, H. Zhang, R. W. Purves, R. W. J. Scott, *Chem. Commun.* **2013**, *49*, 276.

[13] L. Z. Gao, J. Zhuang, L. Nie, J. B. Zhang, Y. Zhang, N. Gu, T. H. Wang, J. Feng, D. L. Yang, S. Perrett, X. Yan, *Nat. Nanotechnol.* **2007**, 2, 577.

[14] K. Zhao, W. Gu, S. S. Zheng, C. L. Zhang, Y. Z. Xian, *Talanta* **2015**, 141, 47.

[15] S. F. Cai, Q. S. Han, C. Qi, Z. Lian, X. H. Jia, R. Yang, C. Wang, *Nanoscale* **2016**, *8*, 3685.

[16] X. J. Wu, T. M. Chen, J. X. Wang, G. W. Yang, *J. Mater. Chem B* **2018**, *6*, 105.

[17] C. F. Jiang, J. Zhu, Z. Li, J. H. Luo, J. S. Wang, Y. Sun, *RSC Adv.* **2017**, *7*, 44463.

[18] Y. Zhang, Z. F. Zhou, F. F. Wen, J. Tan, T. Peng, B. Q. Lou, H. G. Wang, S. X. Yin, *Sensor Actuat. B-Chem*. **2018**, *275*, 155.

[19] L. Han, C. C. Li, T. Zhang, Q. L. Lang, A. H. Liu, *ACS Appl. Mater. Interfaces* **2015**, *7*, 14463.

[20] Z. F. Hu, Z. C. Dai, X. W. Hu, K. Y. Chen, C. H. Gao, X. W. Zheng, Y. Q. Yu, *Anal. Methods* **2019**, *11*, 677.

[21] Q. Liu, P. Chen, Z. Xu, M. Chen, Y. Ding, K. Yue and J. Xu, *Sensor Actuat. B-Chem*. B, **2017**, *251*, 339
